# The Ovary as a Target Organ for New Generation Bisphenols Toxicity

**DOI:** 10.3390/toxics13030164

**Published:** 2025-02-26

**Authors:** Paulina Głód, Joanna Smoleniec, Weronika Marynowicz, Justyna Gogola-Mruk, Anna Ptak

**Affiliations:** 1 Laboratory of Physiology and Toxicology of Reproduction, Institute of Zoology and Biomedical Research, Faculty of Biology, Jagiellonian University, Gronostajowa 9, PL30387 Cracow, Poland; paulina.glod@doctoral.uj.edu.pl (P.G.); joanna.smoleniec@doctoral.uj.edu.pl (J.S.); weronika.marynowicz@doctoral.uj.edu.pl (W.M.); justyna.gogola@uj.edu.pl (J.G.-M.); 2 Doctoral School of Exact and Natural Sciences, Jagiellonian University, Prof. St. Łojasiewicza St 11, PL30348 Cracow, Poland

**Keywords:** bisphenols, ovary, reproductive disorders, fertility, follicular fluid, endocrine-disrupting chemicals, oogenesis, folliculogenesis, steroidogenesis, steroid receptor

## Abstract

Bisphenols (BPs) are a group of organic compounds used extensively in plastics, coatings, and epoxy resins; they have been of concern recently due to their endocrine-disrupting effects. Among these, bisphenol A (BPA) is the most studied. Regulatory measures, such as the ban on BPA use in baby bottles by the European Union and its restricted use in thermal paper, reflect the growing awareness of the health risks of BPA. To mitigate these risks, analogs such as bisphenol S (BPS), bisphenol F (BPF), and others (BPAF, BPAP, BPB, BPP, BPZ) have been developed as alternatives. Despite their intended safety, these analogs have been detected in environmental media, including indoor dust and thermal receipt paper, as well as in human biological samples. Studies report their presence in urine at levels comparable to BPA, with BPS and BPF found in 78% and 55% of samples, respectively. In addition, BPs have been found in human follicular fluid (FF) at concentrations that could exert some paracrine effects on ovarian function and reproductive health. With the increased global production of BPs, occupational exposure and environmental contamination also increase. This review summarizes what is currently known about the effects of BPs on the ovary and the mechanisms by which PBs exert ovarian toxicity, with a particular focus on oogenesis, folliculogenesis, and steroidogenesis. Further, this review emphasizes their influence on reproductive functions and the need for further biosafety evaluations.

## 1. Introduction

**Figure 1:**
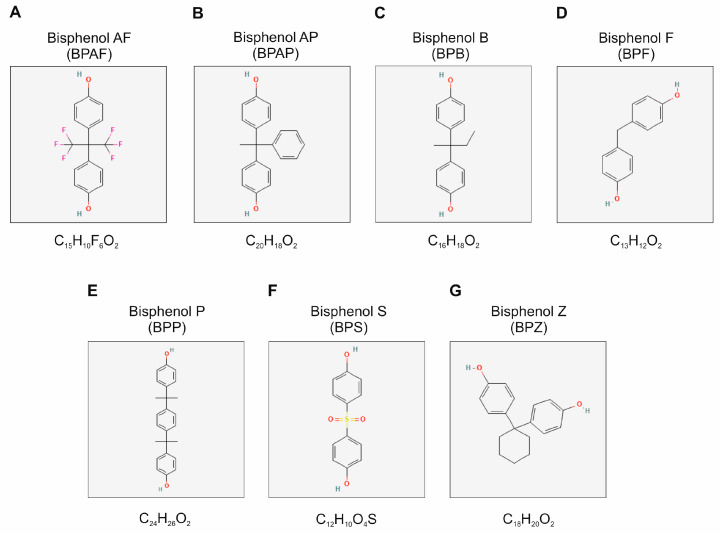
Chemical structure and molecular formula of bisphenol AF (**A**), bisphenol AP (**B**), bisphenol B (**C**), bisphenol F (**D**), bisphenol P (**E**), bisphenol S (**F**), and bisphenol Z (**G**). The structure and molecular formula of each compound was downloaded from the PubChem database [17].

Bisphenols (BPs) are a class of organic compounds used by industries in the manufacturing of plastics, coatings, and epoxy resins (Figure 1). Recently, BPs have come into the spotlight owing to their endocrine-disrupting potential. Among these, the most widely studied is bisphenol A (BPA). Concerns regarding the safety of BPA have resulted in the tightening of regulatory measures against its use. For instance, in 2011, the use of BPA in baby bottles was banned by the European Union [Commission Directive 2011/8/EU [1]]. In addition, the European Food Safety Authority, in 2015, reassessed BPA exposure and toxicity, which decreased TDI from 50 to 4 μg/kg body weight/day (kg/bw/day). By December 2016, BPA was listed in the restricted substances and its use in thermal paper was restricted to less than 0.02% by weight from January 2020 onwards [Regulation 2016/2235 [2]]. In April 2023, experts of EFSA set a tolerable daily intake of 0.2 ng/kg/bw/d replacing the temporary TDI of 4 μg/kg/bw/day. The TDI is roughly 20,000 times lower than before. Due to health concerns, “greener” analogs were developed as alternatives to BPA.

These analogs, such as bisphenol S (BPS), F (BPF), AF (BPAF), AP (BPAP), B (BPB), P (BPP), and Z (BPZ) are widely applied in various fields. However, their occurrence in environmental media across regions indicates large-scale, global contamination. As an example, studies have found these analogs in indoor dust [3,4], in thermal receipt paper [5,6], and in human urine with exposure comparable to levels of BPA [7,8,9,10,11,12]. For example, BPF and BPS were detected in 55% and 78% of urine samples from non-occupationally exposed adults, respectively, indicating their omnipresence [13]. BPA is a known contaminant of concern for female reproductive health and its effects have been linked to follicular fluid (FF) levels [14]. Toxicants concentrations in FF are particularly important as it provides a microenvironment for oocyte development. Non-physiological factors present in the FF may therefore cause a disturbance in ovarian follicle homeostasis, directly affecting ovarian functions. Consequently, the presence of BPs other than BPA in human FF indicates the possibility of their effects on the ovary. The concentrations of BPS and BPF in FF range from about 10 nM to 22 nM, thus indicating a potential for paracrine effects in ovarian microenvironments [15,16].

Since the global production of BPs is on the increase, occupational exposure and environmental contamination are also increasing; therefore, more research should be conducted on their toxicological safety. The aim of the present review is to synthetize and evaluate information on BPs action in the ovary as a target organ for these compounds, pointing out their influence on the main reproductive functions.

## 2. Methodology

### 2.1. Search Strategies

We conducted a literature search in PubMed (https://www.ncbi.nlm.nih.gov/pubmed, accessed on 15 October 2024), Google Scholar (https://scholar.google.com/, accessed on 15 October 2024), Web of Science (https://www.webofscience.com, accessed on 15 October 2024), Scopus (https://www.scopus.com, accessed on 15 October 2024), and ScienceDirect (https://www.sciencedirect.com, accessed on 15 October 2024).

The BPs taken for the literature analysis were BPS, BPF, BPB, BPZ, BPP, BPAF, and BPAP. To write each paragraph, the following keywords were used in combination with each BP: ‘oogenesis’, ‘folliculogenesis’, ‘follicle’, ‘oocyte’, ‘steroid’, ‘steroidogenesis’, ‘progesterone’, ‘estradiol’, ‘corpus luteum’, ‘blood serum concentration’, ‘serum’ ‘follicular fluid concentration’, ‘bisphenol analogs follicular fluid’, ‘urine concentration’, ‘bisphenol analogs in ovary’, impact on ovary’, ‘oocyte’, ‘granulosa cells’, ‘cumulus cells’, ‘COC’, ‘oocyte maturation’, ‘ROS’, ‘infertility’, ‘PCOS’, ‘Endometriosis’, ‘estrogen receptor’, ‘androgen receptor’, ‘insulin-like growth factor 1 receptor’, ‘membrane receptor G-protein coupled receptor 30’, ‘follicle-stimulating hormone receptor’, and ‘luteinizing-hormone receptor’.

Our search identified 1950 studies, of which 96 were considered relevant in terms of the inclusion criteria. The articles included were research studies that measured the levels of BPs in blood, follicular fluid, and urine, studies that investigated the effects of BPs on basic ovarian function, i.e., oogenesis, folliculogenesis, and steroidogenesis, and studies that investigated the ability of BPs to bind and mediate their function through receptors that are crucial for proper ovarian function. We included studies that assessed the association between BPs and ovarian disfunctions and studies that explained the mechanisms by which BPs may affect disorders of the female reproductive system. The selected articles were published between 2009 and 2024. The exclusion criteria were non-original reports, pilot studies, conference abstracts, commentaries, editorials, and articles applied to non-mammals and non-vertebrates. Additionally, review articles were also screened to refine and consolidate relevant information. The findings were limited to those published in English.

### 2.2. Receptor Predictions

To investigate the probability of BPs binding to receptors expressed in ovarian tissue, we used the Endocrine Disruptome tool. Endocrine Disruptome is an open-source, user-friendly, and web-based prediction tool that runs on the Docking interface for Target Systems (DoTS) and uses AutoDock Vina in the background to perform dockings. It uses molecular docking to predict the binding of compounds to 14 different nuclear receptors: the androgen receptor, estrogen receptors α and β, the glucocorticoid receptor, liver X receptors α and β, the mineralocorticoid receptor, peroxisome proliferator-activated receptors α, β/δ, and γ, the progesterone receptor, the retinoid X receptor α, and thyroid receptors α and β. The parameter is the area under the curve (AUC) under the receiver operating characteristic (ROC) curve. Three thresholds were set per structure to allow classification into 4 probability binding classes: red, orange, yellow, and green. Sensitivity (SE) was used for threshold calculations to obtain corresponding docking scores. Class “red” corresponds to SE < 0.25 and indicates a high probability of binding, two intermediate classes “orange” (0.25 < SE < 0.50) and “yellow” (0.50 < SE < 0.75) indicate a medium probability of binding, and class “green” (SE > 0.75) corresponds to a low probability of binding. The docking scores of 6 BPs (using SMILES code) were calculated. Our analyses concentrated on predicting binding to three nuclear receptors with significant functions in ovarian tissue: the androgen receptor and the estrogen receptors α and β. The compounds have a molecular weight lower than 600 g/mol, which is in line with the accuracy of the predictions of this tool. More details for the binding prediction tool are described in a research paper published by Kolšek et al. [18].

## 3. BPs Levels in Human Body Fluids

The occurrence of BPA analogs in different environmental samples has been well studied; however, there is still very little information on the presence of BPs in different human body fluids, especially follicular fluid and in female reproductive tissues [19,20]. Here, we collected the most crucial and recent findings connected to bisphenol analog detection in human blood serum, follicular fluid, and urine; this is presented in Table 1, Table 2 and Table 3.

### 3.1. Blood

The quantification of bisphenol concentration in blood serum is considered a good parameter for biomonitoring exposure. Differential measurements for free BPs as well as the metabolite form with glucuronide or sulfate conjugates have been reported [21,22]. Although BPs can be metabolized and excreted, measuring their concentration in blood and other body fluids helps to assess the level of real-time exposure [23].

BPS was detected in the serum of Polish women at a concentration of 1.135 ng/mL, being one of the most frequent analogs (detection rate = 72%) [24]. Other results showed detection rates of 72.4%, 92.9%, 76%, and 56% in pregnant women, with serum concentrations at the levels of 0.113 ng/mL, 0.007 ng/mL, 0.035 ng/mL, and 0.150 ng/mL, respectively [25,26,27]. For BPF, the serum concentrations were <LOD–0.446 ng/mL, 0.039 ng/mL, 0.115 ng/mL, and 0.062 ng/mL in samples collected from the elderly population living near an e-waste recycling facility. The detection rates were 20.4%, 87.8%, 65%, and >65%, respectively [24,25,26,28]. BPAF was detected in 33.1% of serum samples from pregnant women in China with a concentration range <LOD–0.404 ng/mL [25]. Other studies showed that the concentration of BPAF was not higher than 0.073 ng/mL, although it was found in 72%, 31%, 90%, and >65% of tested blood samples, respectively [26,27,28]. BPB was detected at high levels in the sera of Italian women with endometriosis, where concentrations ranged from 0.88 to 11.94 ng/mL, but not in healthy patients, suggesting its association with reproductive disorders [29]. BPB has also been detected at <LOD–2.52 ng/mL in pregnant women and 0.118 ng/mL with a detection rate of over 27% [25,26]. BPP was detected at concentrations of 1.386 ng/mL, 0.142 ng/mL, and <LOD–0.041 ng/mL, with detection rates of 94.3%, 66.3%, and 4.4%, respectively [24,25,26]. BPZ was rarely detected in serum samples, but two articles showed detection rates of 45.3% and 20.4% at concentrations of 0.235 ng/mL and <LOD–0.108 ng/mL, respectively [24,25]. Very little is known about BPAP detection in serum; however, the compound was found in 44.8% of serum samples from pregnant women from China [25]. Moreover, in red blood cell samples, the fraction of BPAP was 9.9% among all detected BPs [27]. Table 1 summarizes recent studies on the detection of bisphenol analogs in blood serum samples.

**Table 1:** Bisphenol detection in blood serum samples. BPS—bisphenol S, BPF—bisphenol F, BPAF—bisphenol AF, BPAP—bisphenol AP, BPB—bisphenol B, BPP—bisphenol P, BPZ—bisphenol Z, LOD—limit of detection, RBC—red blood cells, N/A—not applicable.

Bisphenol	Sample Type	Detection Frequency [%]	Median [ng/mL]	Range [ng/mL]	Country	Additional Information	Detection Form	Reference
BPAF	serum	33.1	-	<LOD–0.404	China	pregnant women	N/A	[25]
	serum	72	0.010	-	China	industrial area	total (free BP + BP-glucuronide)	[26]
	serum	>65	0.0074	-	China	recycling facility area	N/A	[28]
	plasma	31	0.073	-	China	-	N/A	[27]
	RBC	90	0.017	-	China	-	N/A	[27]
BPAP	serum	44.8	-	<LOD–0.475	China	pregnant women	N/A	[25]
	RBC	9.9	0.013		China	-	N/A	[27]
BPB	serum	41.4	-	<LOD–2.52	China	pregnant women	N/A	[25]
	serum	27.5	0.118	-	China	industrial area	total (free BP + BP-glucuronide)	[26]
	serum	27	5.15 ± 4.16	0.88–11.94	Italy	women with endometriosis	N/A	[29]
BPF	serum	20.4	-	<LOD–0.446	China	pregnant women	N/A	[25]
	serum	87.8	0.039	-	China	industrial area	total (free BP + BP-glucuronide)	[26]
	serum	65	0.115	0.052–0.845	Poland	-	N/A	[24]
	serum	>65	0.062	-	China	recycling facility area	N/A	[28]
BPP	serum	4.4	-	<LOD–0.041	China	pregnant women	N/A	[25]
	serum	94.3	1.386	-	China	industrial area	total (free BP + BP-glucuronide)	[26]
	serum	66.3	0.142	0.057–0.917	Poland	-	N/A	[24]
BPS	serum	72.4	0.113	<LOD–141	China	pregnant women	N/A	[25]
	serum	92.9	0.007	-	China	industrial area	total (free BP + BP-glucuronide)	[26]
	plasma	56	0.150	-	China	-	N/A	[27]
	RBC	76	0.035	-	China	-	N/A	[27]
	serum	72	1.135	0.073–4.844	Poland	-	N/A	[24]
BPZ	serum	20.4	-	<LOD–0.108	China	pregnant women	N/A	[25]
	serum	45.3	0.235	0.053–1.415	Poland	-	N/A	[24]

### 3.2. Follicular Fluid

Follicular fluid (FF) is found in the ovarian follicles surrounding the oocyte, where it plays a crucial role in the development, growth, and maturation of the oocyte. It is necessary for the process of ovulation and fertilization as it provides a microenvironment for the oocyte and surrounding granulosa cells by interacting with systemic factors [30]. FF is derived from peripheral plasma and its composition is highly dependent on the granulosa cells within the follicle [31]. While BPs have been detected in blood, little is known about their presence in FF. Given the potential infertility effects of BPs in women, quantifying their presence in female reproductive organs and fluids could be a potential way to investigate the relationship between BPs levels and reproductive disorders [32,33].

Direct studies of the presence of bisphenol analogs in human reproductive tissues such as the ovary are lacking. However, evidence for their presence in follicular fluid indicates that it is a good marker of local BPs concentration and suggests the potential for accumulation and effects on ovarian tissues. Here, we present recent research on the detection of bisphenol analogs in follicular fluid, summarized in Table 2.

BPS was detected in the FF of women who underwent IVF procedure. The average content of BPS was 5.13 ng/mL, exceeding concentrations found typically in FF samples [15]. Additionally, the glucuronide form of BPS was also detected in a concentration of 0.212 ng/mL [34]. There is little to no evidence of BPF presence in FF, although its glucuronide form was detected in follicular fluid samples in a concentration of 0.130 ng/mL [34]. In the FF from women undergoing IVF procedure from China, BPAF was detected in 50.3% of samples in a concentration of 0.01 ng/mL [35]. In the same study, Li et al. showed concentrations of BPF, BPB, BPAP, BPP, and BPZ below the limit of detection (LOD = 0.002 ng/mL), while in Israel BPB was detected in 39% of FF samples, in a concentration of 0.15 ng/mL [16]. The glucuronide form of BPAF was not detected among tested samples [34] and no information was found about any form of BPP and BPZ concentration above LOD in FF samples.

**Table 2:** Bisphenol detection in follicular fluid samples. BPS—bisphenol S, BPF—bisphenol F, BPAF—bisphenol AF, BPAP—bisphenol AP, BPB—bisphenol B, BPP—bisphenol P, BPZ—bisphenol Z, LOD—limit of detection, N/A—not applicable.

Bisphenol	Sample Type	Detection Frequency [%]	Median [ng/mL]	Range [ng/mL]	Country	Additional Information	Detection Form	Reference
BPAF	follicular fluid	50.3	0.01	<LOD–0.38	China	IVF patients	total (free BP + BP-glucuronide)	[35]
	follicular fluid	n.d.	n.d.	n.d.	France	-	BP-glucuronide	[34]
BPAP	follicular fluid	4.2	<LOD	<LOD	China	IVF patients	total (free BP + BP-glucuronide)	[35]
BPB	follicular fluid	39	0.15	0.00–2.83	Israel	IVF patients	N/A	[16]
	follicular fluid	12.1	<LOD	<LOD	China	IVF patients	total (free BP + BP-glucuronide)	[35]
BPF	follicular fluid	1.4	0.130	0.036–0.277	France	-	BP-glucuronide	[34]
	follicular fluid	17.9	<LOD	<LOD–0.02	China	IVF patients	total (free BP + BP-glucuronide)	[35]
	follicular fluid	3	<LOD	<LOD–1.81	Israel	IVF patients	N/A	[16]
BPP	follicular fluid	4.8	<LOD	<LOD	China	IVF patients	total (free BP + BP-glucuronide)	[35]
BPS	follicular fluid	20	-	0.86 ± 0.13	Spain	-	N/A	[36]
	follicular fluid	100	5.13	0.69–12.16	Czech Republic	IVF patients	total (free BP + BP-glucuronide)	[15]
	follicular fluid	13	0.212	0.025–1.654	France	-	BP-glucuronide	[34]
	follicular fluid	54	0.02	<LOD–0.06	China	IVF patients	total (free BP + BP-glucuronide)	[35]
BPZ	follicular fluid	11.6	<LOD	<LOD	China	IVF patients	total (free BP + BP-glucuronide)	[35]

### 3.3. Urine

The measurement of total bisphenol concentration in urine has been the most widely used method for assessing exposure to these chemicals. Urine concentration can be further compared to blood and FF concentration data to analyze the level of BPs that are not excreted with urine, thus indicating accumulation in other human body fluids. Here, we present several findings concerning total bisphenol concentrations in human urine (Table 3).

BPS was detected in the urine of Dutch women during pregnancy, showing higher concentration and detection frequency in early pregnancy (0.35 ng/mL; 68.1%) than in mid-pregnancy (0.24 ng/mL; 29.1%) [37]. Moreover, pregnant women from South Korea had similar BPS amounts (0.10 ng/mL; 66.1%) to pregnant women from USA (0.09–0.11 ng/mL; 51–57%) [38,39]. In the urine of volunteers from Belgium, USA, and Poland, BPS was detected in 83% (0.12 ng/mL), 92.3% (0.58 ng/mL), and 10.3% of samples, respectively [40,41,42]. As for BPF, studies showed the detection rate of 40.4% and 84.4% with concentrations of 0.58 ng/mL and 0.20 ng/mL, respectively, in the urine of pregnant women [37,38]. BPF was detected most frequently (97%; 0.14 ng/mL) among Belgian adolescents [40], while in USA and Poland BPF was found in 56.5% (0.41 ng/mL) and 19.9% of samples, respectively [41,42]. A study among people exposed to a BPAF manufacturing area showed a BPAF detection rate of <30% and a mean concentration of 0.018 ng/mL [11]. In a study including a Saudi Arabian population, BPAF, BPAP, BPB, BPP, and BPZ were detected in concentrations of 1.10 ng/mL, 0.21 ng/mL, 0.12 ng/mL, 0.16 ng/mL, and 0.10 ng/mL, respectively, proving a general occurrence of BPA analogs in urine [43]. Another study analyzing urine samples from a group of pregnant women identified BPAF, BPAP, and BPP in concentrations of 0.10 ng/mL, <0.02 ng/mL, and <0.02 ng/mL, with detection rates of 100%, 47%, and 7%, respectively [44]. Additionally, among a Portuguese population, BPB was detected at concentrations of 0.21–1.15 ng/mL in 10% of participants [45].

**Table 3:** Bisphenols detection in urine samples. BPS—bisphenol S, BPF—bisphenol F, BPAF—bisphenol AF, BPAP—bisphenol AP, BPB—bisphenol B, BPP—bisphenol P, BPZ—bisphenol Z, LOD—limit of detection, * geometric mean instead of median.

Bisphenol	Sample Type	Detection Frequency [%]	Median [ng/mL]	Range [ng/mL]	Country	Additional Information	Detection Form	Reference
BPAF	urine	-	1.10	-	Saudi Arabia	-	total	[43]
	urine	<30	0.018 *	<LOD–0.173	China	BPAF manufacturing plant area	total	[11]
	urine	100	0.10 *	-	China	pregnant women	total	[44]
BPAP	urine	-	0.21	-	Saudi Arabia	-	total	[43]
	urine	47	<0.02 *	-	China	pregnant women	total	[44]
BPB	urine	-	0.12	-	Saudi Arabia	-	total	[43]
	urine	10	0.68	0.21–1.15	Portugal	-	total	[45]
BPF	urine	40.4	0.58	-	Netherlands	early pregnancy	total	[37]
	urine	84.4	0.20 *	-	South Korea	pregnant women	total	[38]
	urine	56.5	0.41	-	USA	-	total	[41]
	urine	97	0.14	-	Belgium	adolescents	total	[40]
	urine	19.9	-	-	Poland	IVF patients	total	[42]
BPP	urine	-	0.16	-	Saudi Arabia	-	total	[43]
	urine	7	<0.02 *	-	China	pregnant women	total	[44]
BPS	urine	68.1	0.35	-	Netherlands	early pregnancy	total	[37]
	urine	29.1	0.24	-	Netherlands	mid-pregnancy	total	[37]
	urine	66.1	0.10 *	-	South Korea	pregnant women	total	[38]
	urine	51–57	0.09–0.11	-	USA	pregnant women	total	[39]
	urine	83	0.12	-	Belgium	adolescents	total	[40]
	urine	92.3	0.58		USA	-	total	[41]
	urine	10.3	-	-	Poland	IVF patients	total	[42]
BPZ	urine	-	0.10	-	Saudi Arabia	-	total	[43]

## 4. BPs Action in the Ovary

The ovary, as the female gonad, is a crucial endocrine organ responsible for reproductive functions. In humans, female germ cells develop during the first trimester of pregnancy, whereas primordial follicles develop between the second and third trimesters. Females are born with an entire lifetime supply of non-proliferating oocytes (primordial follicles) that survive for around 50 years [46]. The dogma of human (mammalian) development is that no new oocytes and follicles are produced during postnatal life—a recently published molecular study of the human ovary has reinforced this dogma [47]. Therefore, environmental factors affecting folliculogenesis can have a detrimental effect on reproductive functions in women.

### 4.1. BPs Action on the Fetal and Neonatal Ovary

Oogenesis begins in the fetal ovaries, when oogonia are developed from primordial germ cells (PGC); this occurs as soon as the development of the embryo progresses, in approximately the 12th week of gestation in women. Oogonia proliferate by mitosis and form primary oocytes that arrest at the prophase stage of the first meiotic division until they are fully grown [48]. Around the time of meiotic arrest, germ cell nests breakdown to initiate follicle formation. Oocytes become surrounded by somatic (pre-granulosa) cells and form primordial follicles [49]. The development of high-quality oocytes during oogenesis is important for proper fertilization and implantation because it impacts early embryonic survival and the establishment and maintenance of pregnancy, as well as fetal development [50]. Follicle formation occurs before birth in humans and immediately after birth in mice [49]. The human ovaries gradually lose follicles both before and after puberty (the beginning of ovulation); beginning with about several million before birth, the ovaries reach the maximum number at birth, which falls to 300–400,000 by puberty, and finally, by the late 40s, there are only a few follicles left. Recent studies suggest that the original calculations of ovary follicle numbers at birth were over-estimated, and the actual figure should be about 2.5 million [51].

BPs have been shown to affect oogenesis and follicle formation during fetal and early postnatal periods. First, BPS maternal exposure in female mice impaired meiosis and oogenesis by increasing the percentage of oocytes enclosed in primordial follicles and reducing the number of antral follicles [52]. Additionally, due to reduced oocyte adhesion in nests, abnormal germ cell nests breakdown, and primordial follicle assembly is observed post-BPS treatment [53]. In another study, BPS led to oocyte over-loss by accelerating the breakdown of germ cell nests and the assembly of primordial follicles in neonates [54]. Furthermore, BPS has been found to decrease the expression levels of early folliculogenesis-related genes [54], further confirming its disrupting effects on oogenesis dynamics. In human studies, BPS has been shown to significantly affect the ovarian reserve [55]. Low doses of BPF affected spindle formation and epigenetic signs that translated into developmental competence damage [56]. Similarly, BPAF has been shown to delay meiosis initiation with the induction of oocyte aneuploidy. The observed impairments were accompanied by gene expression changes in fetal premeiotic germ cells, causing oogenesis defects [57]. The underlying mechanism for the observed changes remains unknown, but current data explain the observations by increased oxidative stress and apoptosis [54,58]. There are no direct data on the effects of BPAP, BPB, BPP, and BPZ on oogenesis.

### 4.2. BPs Action in Adult Ovary

#### 4.2.1. Folliculogenesis and CL Formation

Folliculogenesis is the process by which immature primordial follicles develop into preovulatory follicles (Graafian follicles). In humans, a primordial follicle takes about 150 days to develop into a preantral follicle (primary) and another 120 days to form an antral follicle (secondary). A number of antral follicles will then “compete” for 14–15 days to become the dominant follicle, which will then undergo ovulation. More than 99% of follicles never enter the preovulatory stage; instead, they undergo atresia through cell apoptosis. After ovulation, granulosa and theca cells undergo luteinization and develop into the corpus luteum (CL). CL is a transient endocrine gland that secretes progesterone (P4) to prepare the uterus for implantation and then pregnancy maintenance. If fertilization fails, the CL undergoes regression by luteolysis, characterized by decreased P4 production. Therefore, folliculogenesis and oocyte health is highly dependent on ovarian and systemic hormones.

In vivo studies have shown a post-BPS treatment decrease in the number of ovarian follicles and antral follicle volume. Depending on dosage, BPS altered ovarian proteome and caused cytoskeletal damage in matured mice oocytes [59]. In rats, prenatal exposure to BPS and BPF caused reduced ovulation characterized by a decreased number of CL [60]. A decrease in antral follicles was also observed for BPF and BPB in adult female rat ovaries [58]. In vitro, BPAF activated the spindle assembly checkpoint, affecting oocyte maturation [61], and decreased the first polar body extrusion [62]. Moreover, BPAF is also able to decrease the numbers and areas of CL [63]. As for BPAP, BPB, and BPZ, similarly to BPAF, these compounds inhibited polar body extrusion, further disrupting spindle assembly and chromosome alignment. Through the impairment of cytoskeletal assembly, the induction of DNA damage, and cell cycle interference, BPAP, BPB, and BPZ were able to negatively affect oocyte maturation in mice [64,65,66].

There is a lack of studies on the in vivo and in vitro effects of BPP on folliculogenesis. Data on the effect of BPs on CL formation are limited to BPS, BPF, and BPAF. The data have been summarized in Table 4.

#### 4.2.2. Steroidogenesis

Steroidogenesis is a process in which important sex steroids such as 17β-estradiol (E_2_), testosterone (T), and P_4_ are formed. The first step of this process, which is also the rate-limiting step, involves the transport of cholesterol from the outer mitochondrial membrane to the inner mitochondrial membrane by the steroid acute regulatory protein (StAR) and other accessory proteins. Once inside the mitochondria, cholesterol is metabolized to pregnenolone, which is then further metabolized in the endoplasmic reticulum to form the final steroid products, including P_4_ and E_2_ via steroidogenic enzymes. The cycling ovary comprises follicles and the CL. During steroidogenesis, antral follicles produce estrogens (principally E_2_) from androgens (androstenedione (A4) and T), whereas the CL produces P_4_. This balance can be disrupted by altering the concentrations of estrogen, androgen, and/or P_4_, or by affecting the expression of steroid hormone receptors. The ovarian steroid hormone receptors include those for estrogen (ERs), androgen (AR), and P_4_ (PR), as well as those for the luteinising hormone (LH) and follicle-stimulating hormone (FSH). The disruption in folliculogenesis or CL formation can lead to reproductive disorders, such as aneuploidy, anovulation, decreased fertility, polycystic ovary syndrome (PCOS), and premature ovarian failure (POF).

Exposure to various BPs, including BPS, BPF, BPAF, BPAP, BPB, BPZ, and BPP, disrupts steroidogenesis across different systems, with effects that are dependent on dose, cell type, and the duration of exposure. In vitro studies have shown a correlation between folliculogenesis impairment and steroidogenesis, where in human luteinized granulosa cells, BPS at 10 µM and 50 µM decreased P_4_ secretion by 16% and 64%, respectively, and 50 µM reduced E_2_ secretion by 46% [67]. Adverse effects were observed in the human non-luteinized granulosa cell line HGrC1, where BPS and BPF at low doses (0.1–100 nM) up-regulated both P_4_ and E_2_ secretion [68]. In bovine granulosa cells, 10 µM BPS reduced P_4_ secretion by 22% (*p* = 0.040) while doubling E_2_ secretion [69]. However, in ovine preantral follicles, 0.1 µM of BPS caused a decrease in E_2_ secretion [70]. Impaired ovine granulosa cell (GC) steroidogenesis has been observed, with disruptions in BPS (50 µM) also tending to reduce steroidogenic enzymes like cytochrome-P450 cholesterol side-chain cleavage (CYP11A1) protein expression by 37% (*p* = 0.0947) without affecting 3β-hydroxysteroid dehydrogenase (3βHSD) and aromatase (CYP19A1) expression [68]. In sheep GCs, BPS at a dose of 0.1 µM reduced P_4_ and E_2_ secretion [69].

**Figure 2:**
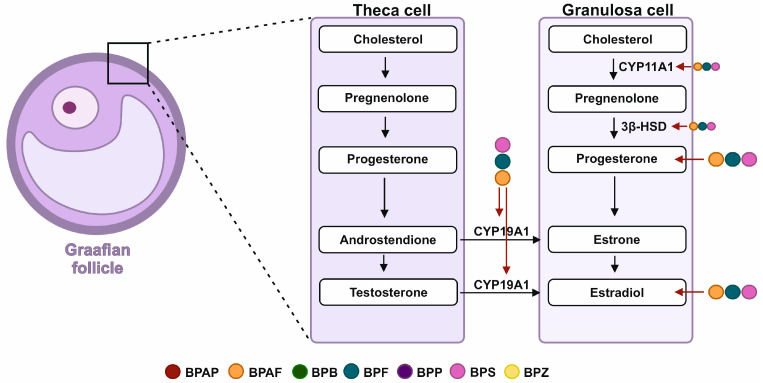
Steroidogenesis is a process occurring in two cell types present in a developing follicle—theca and granulosa cells (GCs). In a series of enzymatic conversions, two main sex steroid hormones, progesterone (P_4_) and 13β-estradiol (E_2_), are secreted. Bisphenols exhibit disrupting abilities for both P_4_ and E_2_ production by GCs and crucial enzyme activity (CYP19A1, CYP11A1, and 3β-HSD). BPS—bisphenol S, BPF—bisphenol F, BPAF—bisphenol AF, BPAP—bisphenol AP, BPB—bisphenol B, BPP—bisphenol P, BPZ—bisphenol Z.

BPS, BPF, and BPAF showed distinct but similarly disruptive effects. In the KGN ovarian tumor granulosa cell line at ≥1 µM, all three BPs decreased progesterone secretion, simultaneously increasing estradiol levels [71]. BPS, BPF, and BPAF negatively affected the expression of key steroidogenic genes, such as StAR (steroidogenic acute regulatory protein), which is involved in cholesterol transport, and disrupted the activity of steroidogenesis enzymes like 3β-hydroxysteroid dehydrogenase (3β-HSD), further impacting steroid hormone synthesis [72,73]. The data have been summarized in Table 4 and Figure 2.

**Table 4:** Bisphenols effect on folliculogenesis, corpus luteum (CL) formation, and steroidogenesis. Progesterone (P4), estradiol (E2), 3β-hydroxysteroid dehydrogenase (3βHSD), aromatase (CYP19A1), cytochrome-P450 cholesterol side-chain cleavage (CYP11A1), steroidogenic acute regulatory protein (StAR); BPS—bisphenol S, BPF—bisphenol F, BPAF—bisphenol AF, BPAP—bisphenol AP, BPB—bisphenol B, BPP—bisphenol P, BPZ—bisphenol Z, nd—no data.

Bisphenol	Folliculogenesis	CL Formation	Steroidogenesis
BPAF	Spindle assembly checkpoint activation in oocyte [61] Decreased first polar body extrusion [62], disrupting spindle assembly and chromosome alignment [64,65,66]	decreased number of CL [64]	Decreased P_4_ and increased E_2_ secretion [71] Decreased expression of StAR and disrupted activity of 3β-HSD [72,73]
BPAP	Inhibited polar body extrusion, disrupted spindle assembly and chromosome alignment, caused DNA damage and cell cycle interference [64,65,66]	nd	nd
BPB	Decreased antral follicle number [58] and inhibited polar body extrusion, further disrupting spindle assembly and chromosome alignment DNA damage and cell cycle interference [64,65,66]	nd	nd
BPF	Decrease in antral follicles number [58]	decreased number of CL [60]	Disruption in P_4_ and E_2_ secretion [68,71] Decreased expression of StAR and disrupted activity of 3β-HSD [72,73]
BPP	nd	nd	nd
BPS	Decreased the number of ovarian follicles and antral follicle volume, altered ovarian proteome and cytoskeletal damage [59]	decreased number of CL [60]	Disruption in P_4_ and E_2_ secretion [67,68,69] Reduced protein expression of CYP11A1 [67] Decreased expression of StAR and disrupted activity of 3β-HSD [72,73]
BPZ	Inhibited polar body extrusion, disrupted spindle assembly and chromosome alignment, Caused DNA damage and cell cycle interference [64,65,66]	nd	nd

#### 4.2.3. BPs Effect on Ovarian Follicle

**Figure 3:**
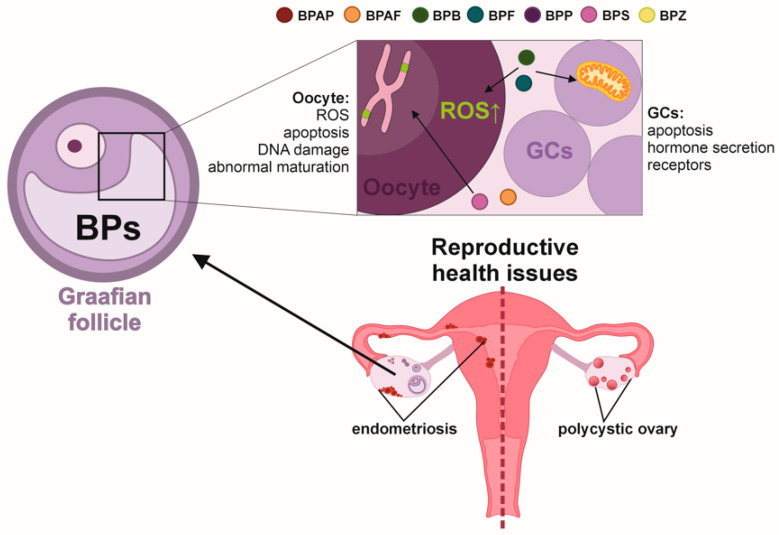
Bisphenols (BPs) detected in follicular fluid can affect Graafian follicles’ microenvironment by causing direct impairments to both oocyte (increased ROS production, apoptosis, DNA damage, abnormal maturation) and granulosa cells (changes in cell cycle, cell death, hormone secretion). This further translates to reproductive health disorders such as endometriosis and polycystic ovary syndrome. BPS—bisphenol S, BPF—bisphenol F, BPAF—bisphenol AF, BPAP—bisphenol AP, BPB—bisphenol B, BPP—bisphenol P, BPZ—bisphenol Z.

The ovarian follicle is a specialized structure within the ovary, which retains important hormonal and reproductive functions. An ovarian follicle consists of single oocyte, granulosa cells (GCs), and theca cells. Specialized GCs, known as the cumulous cells (CCs), surround the oocyte. CCs are a part of cumulus–oocyte complex (COC), crucial for providing nutrients and hormonal support for oocyte development. GCs and theca cells, which surround the follicle, produce hormones like E_2_ and P_4_, and facilitate nutrient and signal exchange to ensure proper follicle development. These interactions are essential for synchronized follicle development and oocyte maturation. However, BPs affect oocytes and follicular support cells such as CCs and GCs (Figure 3).

Spindle abnormalities and chromosome misalignment in bovine oocytes were observed after exposure to low doses of BPS, affecting meiotic progression and spindle morphology [74]. Similarly, BPS exposure disrupted oocyte maturation, decreased cumulus cell expansion, and induced cytoskeletal disruption in pig oocytes [75]. Furthermore, BPS affects the expression of Anti-Müllerian Hormone (AMH) and its receptor during bovine oocyte maturation and early embryo development [76]. Also, BPS affects connexin 37 expression in bovine cumulus cells, which is crucial for cell communication between cumulus cells and oocytes [77]. BPS treatment led to an altered cell cycle, altered estrogen receptor expression, and disrupted cumulus cell expansion in porcine oocytes [75]. A dose of 0.05 mg/mL BPS significantly increased DNA fragmentation at several stages during embryogenesis and altered AMH and its receptor (AMHRII) expression in bovine oocytes, cumulus cells, and Cumulus–Oocyte Complexes (COCs) [76]. In sheep GCs, BPS has also been reported to decrease maturation rate in oocytes [78]. Moreover, chronic low BPS exposure through diet impairs in vitro embryo production parameters according to metabolic status in ewes [79]. As for BPF, its exposure causes oxidative stress and DNA damage in oocytes, which affects their developmental competence and reduces reproductive capacity. Higher doses of BPF (up to 500 µM) induce apoptosis in oocytes, blocking the progression of meiotic maturation by a reduced rate of first polar body extrusion. Furthermore, exposure to 300 µM BPF significantly increases oxidative stress in mouse oocytes, as measured by an increase in reactive oxygen species (ROS) levels, which can damage cellular components and impair oocyte function [80]. In bovine CCs and oocyte complexes, BPF exposure at concentrations ranging from 0.05 mg/mL to 0.5 mg/mL led to oxidative stress and altered the expression of key antioxidant enzymes such as SOD1, SOD2, GPX1, GPX4, and CAT [81]. Additionally, BPF exposure at concentrations of 0.5 µg/mL and 50 µg/mL induces apoptosis in bovine granulosa cells through the intrinsic mitochondrial pathway [82]. BPAF exposure inhibits oocyte maturation in mice, with concentrations of 50 µg/mL and 100 µg/mL and causes a sharp decrease in the number of oocytes reaching maturity. BPAF exposure also leads to cell cycle arrest through the activation of the spindle assembly checkpoint, resulting in abnormal spindle assembly and chromosome alignment [61]. Additionally, BPAF exposure increases oxidative stress and DNA damage in mice oocytes, negatively affecting their maturation [62]. In bovine GCs, BPAF exposure induces apoptosis through the intrinsic mitochondrial pathway, reducing cell viability and increasing the expression of apoptotic genes [82].

#### 4.2.4. BPs and Ovarian Health

With the ability to mimic estrogen, BPs can interfere with hormonal regulation, impacting various aspects of women’s reproductive health. This chapter delves into the associations between BPs exposure and critical conditions such as Polycystic Ovary Syndrome (PCOS), endometriosis, and complications during pregnancy and childbirth. The findings emphasize the need for heightened awareness and preventive strategies to mitigate exposure to these harmful chemicals, safeguarding the health of women and future generations.

PCOS is the most common endocrine disorder among women of reproductive age, with a global prevalence ranging between 5% and 21% [83]. Research has shown that BPs, such as BPAF, BPS, and BPZ, are associated with an increased risk of developing PCOS [84,85]. A study found a statistically significant relationship between the presence of BPAF (aOR = 1.07; 1.02–1.13), BPS (aOR = 1.18; 1.10–1.25), and BPZ (aOR = 1.15; 1.08–1.22) in urine samples and the risk of developing PCOS [86]. In addition, mixed exposure to seven bisphenol analogs was found to be positively associated with the odds of PCOS (adjusted odds ratio = 1.26; 1.12–1.45), primarily driven by BPS (weight = 0.51), BPZ (weight = 0.26), and BPAF (weight = 0.23) [86]. Women who were overweight or obese tended to have a stronger association between bisphenol analogs and PCOS than normal-weight women [87].

Another enigmatic reproductive disorder is endometriosis, which, according to the World Health Organization (WHO), affects 10% of women at reproductive age a year. BPAF has been shown to influence endometriosis through inducing endometriosis lesion growth [87].

Additionally, BPs, including BPS, and BPF, have notable impacts on pregnancy and childbirth. A cohort study from Shenyang, China, demonstrated that BPS concentrations were significantly associated with a seven-fold higher risk of diminished ovarian reserve. Additionally, the study found a significant negative association between BPS levels and Anti-Müllerian Hormone (AMH) levels (*p* = 0.010) [55]. Exposure to BPS has also been correlated with oxidative stress and homeostatic imbalance, which are linked to recurrent spontaneous miscarriage [88]. Furthermore, BPS exposure during the first trimester has been statistically significantly associated with an increased risk of developing gestational diabetes mellitus [89]. It was also found that first trimester exposure to BPS was associated with larger fetal head circumference and fetal weight, and both BPS and BPF in the first trimester were linked to a lower risk of being born small for gestational age [90]. Elevated levels of BPS in maternal urine, particularly during the first trimester, are associated with larger fetal head circumference, greater weight, and a decreased risk of being small for gestational age at birth [90]. In addition, higher concentrations of BPF were found to be positively linked to an increased risk of preterm birth. Prenatal exposure to bisphenol mixtures, including BPF, may elevate the likelihood of preterm birth [91].

Maternal exposure to BPS or BPF can lead to their accumulation in the fetal compartment, causing chronic exposure and potentially hindering normal fetal growth and development. The potential mechanisms through which these BPs exert their effects include impaired placental function and development, gene expression dysregulation, hormonal imbalances, immune response disturbances, induced inflammation, and oxidative stress [92]. Moreover, there is evidence describing environmental exposure to bisphenol analogs and unexplained recurrent miscarriage (URM). The concentrations of six bisphenol analogs (BPA, BPAF, BPAP, BPB, BPP, and BPS) were measured in the urine samples. The mixed exposure of six bisphenol analogs was positively associated with the risk of URM (adjusted odds ratio (aOR) = 1.25; 1.11–1.42), which was mainly driven by BPAP (60.1%), BPAF (25.1%), and BPA (14.8%). After age stratification, the risks tended to be higher in women aged 30 years or older compared to women < 30 years [93].

### 4.3. Molecular Mechanism of BPs Action

#### 4.3.1. Sex Steroid Nuclear Hormone Receptors

There are several pathways of actions for BPs in the ovary; such compounds mainly act by interference in the activity of nuclear steroid hormone receptors like ERs, AR, and PR. The main affected pathways include ERs including estrogen receptor alpha (ERα) and estrogen receptor beta (ERβ). These receptors are critical mediators of the biological effects of estrogen in the ovary and are key to maintaining ovarian GCs differentiation, follicle and oocyte growth and development, and ovulatory function.

BPS, BPB, BPF, and BPAF have comparable or superior estrogenic agonist potencies on human ERα and ERβ [94,95]. The prediction analysis of the probability of binding to the nuclear receptor suggests that BPAF has a high probability of binding to ERβ as an agonist and a medium probability of binding to ERβ as an antagonist and to ERα as both an agonist and as an antagonist (Table 5). The literature further confirms those findings, as the compound has been shown to induce apoptosis via the ERβ pathway in the human GC line KGN [96]. BPAP exhibited a high probability of binding to ERα as an agonist and to ERβ as an antagonist, and a medium probability of binding to ERα as an antagonist ([97], Table 5). Similarly, BPB has been established as a bifunctional ERα agonist and ERβ antagonist [97]; BPF demonstrated activity towards ERα and ERβ, with a stronger affinity to ERα [98]. Furthermore, the analysis of the predicted binding probabilities of nuclear receptors showed that BPP binds with high probability to ERβ as an antagonist and with medium probability to ERα as an antagonist. Furthermore, BPZ binds with a high probability to ERα as an agonist and ERβ as an antagonist, and with a medium probability to ERα as an antagonist and ERβ as an agonist (Table 5).

There is a gap in the literature regarding the effects of BPs through ER strictly on the ovary. A few studies have shown the BPZ activation of ERα and Erβ but focused mainly on breast cancer cells, which confirmed its ability to act through those pathways, as shown by the probability of binding analysis [97,99].

Moreover, the prediction analysis shows that BPs can affect androgen receptor (AR) signaling. The AR is present in all three components of the ovarian follicle, namely the GCs, theca, and oocyte [100,101]. The predictive analysis of the probability of binding to NR indicates that BPAF has a high probability of binding and BPF, BPB, BPZ, and BPS have a medium probability of binding to AR as an antagonist (Table 5). The prediction did not indicate a BPAP affinity for AR; the literature also lacks in the description of BPAP action in the ovary through this pathway. There is a comprehensive study on the ability of binding to AR by BPAP, where the lack of affinity is further confirmed [102]. There is only one report on a non-ovarian model, where BPAP down-regulated AR expression [103].

#### 4.3.2. Membrane Ovarian Receptors

Crucial for the process of folliculogenesis and ovulation, and, therefore, for overall reproductive health, are two transmembrane receptors: the follicle-stimulating hormone receptor (FSHR) and luteinizing-hormone receptor (LHR); these are responsible for mediating the action of the follicle-stimulating hormone (FSH) that regulates follicle growth and survival during folliculogenesis [104,105] and the luteinizing hormone (LH) responsible for proper ovulation during menstrual cycle and the regulation of progesterone secretion in CL [106].

The literature suggests that BPs can have an indirect influence on FSHR and LHR as BPAF and BPS were able to alter the hypothalamic–pituitary–gonadal (HPG) axis function, resulting in impaired LH and FSH production. Additionally, BPS has been shown to directly reduce the action of both FSHR and LHR [73]. For other mentioned BPs, despite known effects on folliculogenesis and overall ovarian health, there is a clear lack of studies discussing the influence of said compounds on FSHR and LHR activation.

The insulin-like growth factor 1 receptor (IGF1R) is another transmembrane receptor that has a role in ovarian function and female fertility, which also involves the regulation of follicle growth and survival as well as GCs differentiation. IGF1R action directly correlates with ER and FSHR, as the literature points out the crosstalk between nuclear ESRs, IGF1R, and the membrane receptor G-protein-coupled receptor 30 (GPR30) receptor [107,108]. Additionally, in vitro studies indicated that in the absence of IGF1R, FSH’s effect on GCs proliferation is diminished [109]. As for the BPs action through IGF1R, only BPS and BPF have been found to affect human GCs steroidogenesis through acting via this pathway [68], suggesting their ability to affect IGF1R; however, further research is necessary to explore any potential interactions. Additionally, BPB has been suggested to mediate its function through the non-classical GRP30 pathway [110], similar to BPS and BPF [111,112].

## 5. BPs as Female Reproductive Toxicants

In 2019, 10 key characteristics (KCs) of chemicals that cause female reproductive toxicity were proposed by Luderer et al.: (1) the alteration of hormone receptor signaling, reproductive hormone production, secretion, or metabolism; (2) genotoxicity; (3) epigenetic alterations; (4) the cause of mitochondrial dysfunction; (5) oxidative stress induction; (6) the alteration of immune function; (7) the alteration of cell signal transduction; (8) the alteration of direct cell–cell interactions; (9) the alteration of survival, proliferation, cell death, or metabolic pathways; and (10) the alteration of microtubules and associated structures [113]. The present review addresses each of the ten to a greater or lesser extent, highlighting three key ones relating to endocrine dysregulation, mitochondrial activity, and general changes in cell biology that negatively affect women’s reproductive health. This review therefore suggests that BPs could potentially be classified as reproductive toxicants.

### Knowledge Gaps and Further Research Needs

According to the aforementioned KCs and the data presented in this study, despite reports on the effects of BPs on reproductive health, the available data are still insufficient for drawing certain conclusions. Firstly, further research is needed to elucidate human exposure to BPs (excluding BPA) with the exact quantification of total and free concentrations in tissues and body fluids. Consequently, metabolic modification effects on the reproductive toxicity of BPs should be further investigated. In order to describe the sources of BP-induced changes, it is necessary to define the activated molecular mechanisms, with specifications on receptor binding and, thus, triggered pathways. Most of the available studies on the toxicity of BPs focus on the effect of exposure to a single compound, whereas the chemicals are detected simultaneously, indicating the need for co-exposure research.

## 6. Conclusions

This review showed the presence of BPs in human blood serum, follicular fluid, and urine, which could indicate their possible roles in risks to reproductive health. Although several BPs were shown to be frequently detected, information on the others remains limited and calls for further studies. Blood and follicular fluid are important media in monitoring exposure and possible health effects, particularly in reproductive concerns, and therefore merit further study regarding their distribution and impact on human health.

BPs seriously affect both ovarian follicle health and whole reproductive function, leading to such disruptions as oxidative stress, the disturbance of hormone levels, and DNA damage. These chemicals disrupt critical processes such as oocyte maturation, cumulus cell expansion, GCs steroidogenesis, and CL formation, leading to diminished fertility and impaired embryo development. This can result in poor oocyte quality and reduced fertility. Understanding these roles and relationships is vital for improving fertility treatments and reproductive health. Moreover, our findings highlight the significant and multifaceted impacts of BPs on pregnancy outcomes and underscore the importance of minimizing exposure to these chemicals to protect maternal and fetal health.

Their effects on ovarian function are mediated primarily via interference with nuclear and non-classical steroid hormone receptor pathways. The main targets are estrogen receptors, including ERα, ERβ, and GPR30, and androgen receptors, which play critical roles in the differentiation of granulosa cells, follicle development, and ovulation. The differences in the binding affinities of various BPs define their specific modes of action, such as ER modulation and AR antagonism.

BPs are further implicated in female reproductive diseases, including PCOS and endometriosis, and may adversely affect pregnancy outcomes and fetal development. Although progress has been made in understanding these mechanisms, significant gaps remain in evaluating the full impact of certain BPs, and further research will be needed to clarify their role in ovarian health and reproductive disorders.

## Figures and Tables

**Table 5:** Probability of BPs (BPAF, BPAP, BPB, BPF, BPP, BPS, BPZ) binding to nuclear receptors based on the Endocrine Disruptome web prediction tool. +++ high probability of binding (class “red”; SE < 0.25), ++ medium probability of binding (class “orange” (0.25 < SE < 0.50), and “yellow” (0.50 < SE < 0.75)). SE—threshold calculations sensitivity [18].

	ERα		ERβ		AR	
	Agonist	Antagonist	Agonist	Antagonist	Agonist	Antagonist
BPAF	++	++	+++	++		+++
BPAP	+++	++		+++		
BPB						++
BPF						++
BPP		++		+++		
BPS						++
BPZ	+++	++	++	+++		++

## Data Availability

No new data were generated for this review.
